# Study on biochemical divergences of the meat and egg of freshwater prawns (*Macrobrachium rosenbergii*)

**DOI:** 10.1002/fsn3.1031

**Published:** 2019-05-07

**Authors:** Thi Mong Tho Nguyen, Tai‐Yuan Chen, Chyuan‐Yuan Shiau, Ying‐Tzu Cheng, Yu‐Wei Chang

**Affiliations:** ^1^ Department of Food Science National Taiwan Ocean University Keelung Taiwan; ^2^ National Agro‐Forestry‐Fishery Quality Assurance Department Ca Mau Vietnam

**Keywords:** biochemical characteristics, egg, freshwater prawn, meat

## Abstract

Aquaculture of freshwater prawn (*Macrobrachium rosenbergii*) is an important fishery sector in Asian countries. The biochemical characteristics including proximate compositions and taste components of cultured freshwater prawns were analyzed for different gender and their egg. Freshwater prawns contained moisture ranging from 77.51% to 78.37%, protein with 19.56 to 19.91%, and low levels of lipid and ash around 0.2 and 1%, respectively. The moisture and protein contents of egg samples (72.82 and 15.61%, respectively) were lower than meat samples, whereas for the lipid content, it was otherwise significantly higher than the meat samples. The male and female meat samples of freshwater prawns were rich in free amino acids (FAAs), namely 1756 to 1725 mg/100 g with arginine, proline, glycine, glutamic acid, lysine, alanine, and taurine being the predominant compounds. On the contrary, the FAA amount of egg (590 mg/100 g) was remarkably lower than meat samples. The ATP‐related compounds in freshwater prawn meat ranged from 8.26 to 8.80 μmole/g, majorly composed of adenosine diphosphate (ADP), adenosine triphosphate (ATP), adenosine monophosphate (AMP), and inosine 5’‐monophosphate (IMP). The ATP‐related values in egg sample (28.33 μmole/g) were significantly higher with IMP (18.48 μmole/g) and AMP (5.44 μmole/g) being the predominant compounds. The pH values of male and female meats were neutral, whereas this value in egg sample was acidic (6.5). Urea content in egg sample was detected at huge value (58.35 μmole/g), whereas it was not detected in meat samples. These findings have established useful information for product developers to utilize the meat and eggs of freshwater prawns. In addition, the assessment of FAAs, ATP‐related compounds, and quality index (pH and urea content) was also established to give fundamental criteria to evaluate the quality of fresh *M. rosenbergii*.

## 1 INTRODUCTION

Freshwater prawns, *Macrobrachium rosenbergii,* belong to the family *Palaemonidae* which includes the brackish and freshwater grass shrimp and the larger river shrimp (Eglal et al., 2011). *Macrobrachium* *rosenbergii* is a commercially important species of crustacean cultured extensively throughout South‐East Asia and plays an important role in the aquaculture and fisheries industry. *Macrobrachium rosenbergii* has always been concerned as a suitable species for aquaculture because it can be grown in both fresh and low salinity waters with good growth and survival rates. In addition, absence of major disease problems, a wide consumer acceptability, and high economic value make this prawn prevalent in the market (Balazs & Ross, 1976). The domestic and international markets of peeled, head‐on, and shell‐on products of the species exist and are gradually expanding all over the world (Lalrinsanga et al., 2014; Ziaei‐nejad, Rafiee, & Shakouri, 2009). Freshwater prawn is currently popular with small‐scale aquaculture farms in Asian countries including Vietnam, Thailand, and Indonesia. It has a potential development for rural aquaculture business. In the meanwhile, considerable employment and income could be generated, thereby bringing prosperity to rural people. Production of freshwater prawns was valued above $1 billion (2009) annually with over 98% of production occurring in Asia (Ziaei‐nejad et al., 2009). Aquaculture of the freshwater prawns *M. rosenbergii* is an emerging industry in the Pacific Island region (Nandlal & Pickering, 2005). Increasing demand and rising prices for seafood are raising the profile of freshwater prawns as an important aquaculture commodity in Pacific Island countries and territories (Nandlal & Pickering, 2005). However, freshwater prawn products are limited in the markets because their quality is degraded quickly through autolytic, microbial, and oxidative spoilage (Haider et al., 2013). Therefore, the engagement in the production of high‐quality products of freshwater shrimps becomes one of the most important tasks. Biometric profiles of freshwater prawns are restrained to limited literatures. In this regard, the objective of the present study was therefore to determine the biochemical characteristics including proximate composition (moisture, protein, lipid, and ash), taste components (free amino acids, dipeptides, and nucleotide‐related compounds), and quality index such as pH, urea, and NH_3 _values. Both the meat samples of freshwater prawns and egg samples collected from gravid females were investigated in order to provide fundamental information for potential applications.

## 2 METHODS AND MATERIALS

### 2.1 Sampling

Freshwater prawns (*M. rosenbegii*) including male, female, and gravid female prawns were used in this study for analyzing their quality and biochemical composition. The sample source, sampling date, weight, length, and condition factor (CF) are listed in Table 1. The CF observed in this study ranged from 1.09 to 1.22, indicating that the prawns were in good condition (Pauly, 1984).

**Table 1:** Description of freshwater prawn samples and condition factor (CF)

Sample names	Source	Sampling date	Weight (g)	Length (cm)	Condition factor (CF)*
Large males	Market	Jul−2015	37.15 ± 6.14	14.72 ± 0.72	1.16 ± 0.13
Small males	Market	Jul−2015	24.24 ± 3.13	13.04 ± 0.61	1.09 ± 0.07
Large females	Market	Jul−2015	38.36 ± 3.54	14.79 ± 0.58	1.19 ± 0.08
Small females	Market	Jul−2015	25.85 ± 2.50	13.26 ± 0.50	1.11 ± 0.12
Gravid females	Fishing farm	Oct−2016	27.21 ± 4.52	13.07 ± 0.88	1.22 ± 0.10

* CF = (Weight × 100)/(Length)^3^.

Alive samples were purchased in July 2015 from the market in Keelung City, Taiwan, for biochemical composition analysis. Prawns were killed by submerging them in a bucket containing crushed ice with water. After that, the prawns were sorted into 2 groups: male and female. Samples from each group were beheaded and peeled to get tail meat which was chopped to prepare for biochemical analyses. Gravid female samples were bought from fishing farm in October 2016 to get the egg for biochemical composition analysis. The samples had in average body weight from 24.24 to 14.79 g and length from 13.04 to 16.28 cm (Table 1).

### 2.2 Analytical method

#### 2.2.1 Proximate composition

The moisture, ash, crude protein, and crude lipid content of the prawn were analyzed by following the methods of AOAC (2005).

#### 2.2.2 Moisture

Moisture content was measured by the weight difference before and after oven‐drying at 105°C for at least 6 hr. The moisture content was calculated by the following formula:

$$\text{Moisture}\;\left(\%\right)=\left(\text{weight loss}/\text{original weight of sample taken}\right)\times 100\left(\%\right).$$

#### 2.2.3 Ash

Ash content was measured by the weight difference before and after ashing furnace at 550°C for at least 6 hr. The ash content was calculated by the following formula:

$$\text{Percentage}\;\left(\%\right)\text{of Ash}=\left(\text{weight of ash}/\text{weight of sample}\right)\times 100\left(\%\right).$$

#### 2.2.4 Crude protein

The method consists of 3 stages: digestion, distillation, and titration.

Digestion: take approximately 2 g (W) sample into the digestion tube, add 5 g catalyst (K_2_SO_4_: CuSO_4_.5H_2_O = 9:1) and 15 ml of concentrated sulfuric acid, and then put it into the digester (380°C) until solution clear.

Distillation: after cooling down the solution, add 70 ml of distilled water and 80 ml 35% NaOH, and then distill the solution with 20 ml 4% boric acid and 2 drops of crude protein indicator within 5 min.

Titration: titrate the solution with 0.1 N sulfuric acid until the solution turns into pale pink color. The amount of consumed sulfuric acid (V) was recorded.

The crude protein content was estimated from the total nitrogen multiplied by nitrogen factor 6.25 as the following formula:

$$\text{Percentage}\;\left(\%\right)\;\text{of proteins}=\left[0.0014\times V\times 6.25\right]/W\times 100\left(\%\right).$$

#### 2.2.5 Crude lipid

Crude lipid content was measured by dried 3 g sample in 105°C oven at least 6 hr and then extracting the lipid with ether in a Soxhlet extractor for 6 hr. After extraction, the fat cup was put into the 105°C oven until constant weight to remove moisture and then cooled down in the desiccator and recorded the extract weight. The crude lipid content was estimated by the following formula:

$$\text{Percentage}\;\left(\%\right)\text{of Lipids}=\left(\text{Weight of the extract}/\text{Weight of sample}\right)\times 100\left(\%\right).$$

#### 2.2.6 Determination of pH value

1 g sample was homogenized with 10 ml distilled water for 1 min. The pH value of the filtrate was directly recorded by using a calibrated glass electrode pH meter for 30–60 s.

#### 2.2.7 Analysis of free amino acid, dipeptides, urea, and ammonia

##### Preparation of extract

The extract of free amino acids, dipeptides, urea, and ammonia was prepared using the method of Konosu, Watanabe, and Shimizu (1974) with some modifications. 20 ml of 7% cold TCA (trichloroacetic acid) was added to 5 g sample and then homogenized by Polytron PT‐3000 homogenizer for 2 min. Following that, the homogenate was centrifuged at 4,000 rpm (4°C) for 20 min. The supernatant was filtered by Advantec Toyo No. 2 filter paper. The precipitate was extracted twice with 7% cold TCA with the same procedure. Filtered supernatants were combined and made up to 100 ml with 7% cold TCA and then stored at −20°C.

For free amino acid analysis, a 40 ml TCA supernatant was mixed with an equal amount of diethyl ether to remove TCA and fat by using Separatory Funnel Shaker for 5 min. This procedure was repeated 5 times to completely remove TCA and fat. Following that, the aqueous solution was evaporated to dryness in a vacuum evaporator at 43°C. The dried matter was diluted with redistilled water and made up to 25 ml and then stored at −20°C.

##### High‐speed automatic amino acid analyzer method

The free amino acids, dipeptide, urea, and ammonia were separated by ion exchange chromatography and analyzed by a Hitachi L‐8900 high‐speed automatic amino acids analyzer with a Hitachi 2,622 SC packed column (4.6 mm × 60 mm). This machine uses the reaction column system to provide the highest sensitivity among the amino acid analyzers using the ninhydrin reaction method. The buffers used were the standard lithium citrate buffers. Postcolumn derivatization with ninhydrin yielded amino acid derivatives, which measured the absorbance at 570 nm and 440 nm. Analytical conditions and procedure were performed according to the manual provided by the manufacturer (Hitachi, Ltd., Tokyo, Japan). Free amino acid, urea, and ammonia levels were estimated in the basis of peak areas of known standard concentrations (Wako, Ltd., Osaka, Japan) using a Hitachi D‐2850 Chromato data processor.

#### 2.2.8 Determination of ATP‐related compounds

##### Preparation of extract

20 ml of 7% cold TCA (trichloroacetic acid) was added to 5 g sample and then homogenized by Polytron PT‐3000 homogenizer for 2 min. Following that, the homogenate was centrifuged at 4,000 rpm (4°C) for 20 min. The supernatant was filtered by Advantec Toyo No. 2 filter paper. The precipitate was extracted twice with 6% cold PCA with the same procedure. The clear filtrates were combined and adjusted to pH 6.5 with 1N or 10N KOH, and then placed in an ice bath for 30 min to precipitate potassium perchlorate. After that, the supernatant was again filtered by Advantec Toyo No. 2 filter paper. Following that, the supernatant was made up to 100 ml with 6% cold PCA (pH 6.5) and then stored at −20°C.

##### High‐performance liquid chromatography (HPLC) method

Adenosine 5’‐triphosphate (ATP), adenosine 5’‐diphosphate (ADP), adenosine 5’‐monophosphate (AMP), inosine 5’‐monophosphate (IMP), inosine (HxR), and hypoxanthine (Hx) in the PCA extract were determined by high‐performance liquid chromatography (HPCL, Hitachi pump L‐2130, Hitachi UV detector L‐2400). Previously, 20 μL PCA extract was filtered through a 0.22‐μm membrane and then injected into a Cosmosil packed column 5C18‐MS (4.6 mm × 250 mm, Nacalai Tesque, Inc., Japan). Two mobile phases used for the separation of nucleotide compounds consisted of eluent A, 50 mm KH_2_PO_4 _(pH 6.5), and eluent B, 50 mm KH_2_PO_4 _(pH 6.5). The gradient elution procedure was similar to the method described by Suwetja, Hori, Miyazawa, and Ito (1989) with a little modification. The flow rate was set to 1.0 ml/min, and the column temperature was held at 25℃. The eluent was monitored by UV absorption at 254 nm. The amounts of nucleotide breakdown compounds were determined by comparing with standards (Sigma). The result was expressed as K values (Saito, Arai, & Matsuyoshi, 1959), calculated by using the following formula:

$$\text{K value}\;\left(\%\right)=\left[\left(\text{HxR + Hx}\right)/\left(\text{ATP}+\text{ADP}+\text{AMP}+\text{IMP}+\text{HxR}+\text{Hx}\right)\right]\times 100\left(\%\right).$$

### 2.3 Statistical analysis

All data were analyzed by using one‐way ANOVA in SPSS (Statistical Package for the Social Science) statistics (2011). Duncan's multiple range test was applied to determine significance of differences between means. A value of *p* < 0.05 was used to indicate significant difference.

## 3 RESULTS AND DISCUSSION

### 3.1 Proximate composition

The proximate composition in percentage of male meat, female meat, and egg of freshwater prawn is shown in Table 2. There were no significant differences in proximate composition of freshwater prawn in different gender. Freshwater prawn contained high moisture and protein with respective values from 77.51 ± 0.48% to 78.37 ± 0.02% and from 19.56 ± 0.26% to 19.91 ± 0.42%, and a very low level of lipid and ash, around 0.2% and 1%, respectively. These proximate compositions are very close to the values of freshwater prawn reported by Gaberz Kirschnik, Viegas, Valenti, and Oliveira (2006), with moisture, protein, lipid, and ash being 78.25%, 18.59%, 0.29%, and 1.35%, respectively. Guevara, Nan, and Shiau (2008) found that white shrimp had 71.1 to 78.9% moisture, 18.6 to 23.2% protein, 0.23 to 0.71% lipid, and 1.39 to 2.92% ash. Freshwater prawn is considered as lean seafood (fat content of less than 5%) (Stansby & Alverson, 1976). The moisture and protein content of egg samples (72.82 and 15.61%, respectively) were lower than meat samples, whereas for the lipid content, it was otherwise significantly higher than the male and female meat samples. The contents of ash in male and female meat recorded were 1.35% and 1.46%, respectively, while this value in egg reaches 2.27 ± 0.13%. Methods for staging eggs have been useful in operating hatcheries for the freshwater prawn *M. rosenbergii*. Ling (1978) described how newly spawned prawn eggs go through a sequence of color changes from orange to dark brown as they mature. Color change during egg development was found to be a useful indicator for staging eggs and predicting hatching red claw crayfish (Yeh & Rouse, 1994).

**Table 2:** Proximate compositions (%) of male meat, female meat, and egg of freshwater prawn

	Male meat	Female meat	Egg
Moisture	78.37 ± 0.02^a^	77.51 ± 0.48^b^	72.82 ± 0.20^c^
Protein	19.91 ± 0.42^a^	19.56 ± 0.26^a^	15.61 ± 0.36^b^
Lipid	0.26 ± 0.04^b^	0.20 ± 0.01^b^	4.06 ± 0.55^a^
Ash	1.35 ± 0.05^b^	1.46 ± 0.21^b^	2.27 ± 0.13^a^

Note

Tests were performed in triplicate; values are means ± standard deviation; mean values in the same column with different superscript letters are significantly different at *p* < 0.05.

### 3.2 Free amino acids and dipeptides

The free amino acids (mg/100 g) of male meat, female meat, and egg of freshwater prawn are revealed in Table 3. Freshwater prawn was rich in free amino acids (FAAs) where there was no significant difference between male and female samples, namely 1756.5 ± 321.8 to 1725.1 ± 223.4 mg/100 g, respectively. In particular, the arginine (from 638.1 ± 81.6 to 663.6 ± 62.6 mg/100 g), proline (from 285.6 ± 119.9 to 329.7 ± 90.6 mg/100 g), glycine (from 163.9 ± 67.7 to 183.0 ± 64.4 mg/100 g), glutamic acid (from 120.3 ± 37.3 to 129.3 ± 25.6 mg/100g), lysine (from 74.3 ± 16.7 to 82.8 ± 5.4 mg/100 g), alanine (from 69.5 ± 11.6 to 75.1 ± 2.4 mg/100 g), and taurine (from 53.7 ± 26.9 to 66.6 ± 25.8 mg/100 g) were the predominant compounds. The FAA amount of egg prawn (590.3 ± 161.98 mg/100 g) was remarkably lower than that of meat samples with the highest values belong to arginine (74.4 ± 0.44), glutamic acid (67.7 ± 14.07 mg/100 g), proline (64.2 ± 30.15 mg/100 g), taurine (42.5 ± 1.74 mg/100 g), alanine (39.1 ± 15.29 mg/100 g), and glycine (31.8 ± 13.08 mg/100 g).

**Table 3:** Free amino acid (mg/100g) of male meat, female meat, and egg of freshwater prawn

FAAs (mg/100 g)	Male meat	Female meat	Egg
Phosphoserine	–*	–	9.4 ± 0.63^a^
Taurine	66.6 ± 25.8^a^	53.7 ± 26.9^a^	42.5 ± 1.74^a^
Aspartic acid	7.3 ± 1.2^ab^	3.6 ± 3.2^b^	11.2 ± 2.62^a^
Threonine	16.0 ± 0.8^a^	19.7 ± 5.9^a^	11.4 ± 3.79^a^
Serine	29.7 ± 6.0^a^	29.9 ± 7.5^a^	11.2 ± 2.82^b^
Glutamic acid	120.3 ± 37.3^ab^	129.3 ± 25.6^a^	67.7 ± 14.07^b^
Sarcosine	14.2 ± 3.6^a^	11.6 ± 8.0^a^	–
Aminoadipic acid	2.4 ± 1.2^a^	1.8 ± 1.9^a^	0.5 ± 0.11^a^
Glycine	183.0 ± 64.4^a^	163.9 ± 67.7^a^	31.8 ± 13.08^b^
Alanine	75.1 ± 2.4^a^	69.5 ± 11.6^a^	39.1 ± 15.29^b^
Citrulline	–	–	2.2 ± 0.11^a^
α‐Aminobutyric acid	–	–	3.0 ± 0.68^a^
Valine	25.4 ± 0.9^a^	27.4 ± 6.1^a^	14.6 ± 5.66^b^
Cystine	–	–	3.8 ± 1.58^a^
Methionine	9.3 ± 1.5^a^	9.9 ± 1.5^a^	2.7 ± 3.13^b^
Cystathionine	21.5 ± 7.3^ab^	24.7 ± 1.0^a^	10.1 ± 8.30^b^
Isoleucine	8.8 ± 1.5^a^	38.2 ± 51.8^a^	9.3 ± 4.33^a^
Leucine	20.8 ± 2.1^a^	22.1 ± 2.8^a^	22.1 ± 9.95^a^
Tyrosine	24.7 ± 0.3^a^	19.2 ± 2.9^a^	28.7 ± 7.70^a^
Phenylalanine	11.7 ± 0.2^a^	14.2 ± 8.9^a^	14.2 ± 4.39^a^
β‐Alanine	3.5 ± 0.0^b^	2.8 ± 0.5^c^	4.7 ± 0.22^a^
β‐Aminoisobutyric acid	2.2 ± 1.6^a^	1.7 ± 1.7^a^	2.6 ± 0.43^a^
γ‐Aminobutyric acid	1.6 ± 1.1^b^	1.5 ± 1.0^b^	18.7 ± 12.03^a^
Tryptophan	2.6 ± 1.3^b^	1.6 ± 1.3^b^	5.7 ± 1.79^a^
Hydroxylysine	–	–	5.6 ± 0.16^a^
Ornithine	2.0 ± 0.0^b^	1.7 ± 1.1^b^	5.8 ± 2.15^a^
Lysine	82.8 ± 5.4^a^	74.3 ± 16.7^a^	33.4 ± 6.21^b^
Histidine	53.8 ± 0.9^a^	53.5 ± 3.3^a^	15.9 ± 2.92^b^
Arginine	638.1 ± 81.6^a^	663.6 ± 62.6^a^	74.4 ± 0.44^b^
Proline	329.7 ± 90.6^a^	285.6 ± 119.9^a^	64.2 ± 30.15^b^
Total	1756.5 ± 321.8^a^	1725.1 ± 223.4^a^	590.3 ± 161.98^b^

Note

Tests were performed in triplicate; values are means ± standard deviation; mean values in the same column with different superscript letters are significantly different at *p* < 0.05.

* Not detected.

Proline is derived from glutamic acid. This is one of the main amino acids our body uses to build collagen (Peterkofsky & Udenfriend, 1965), which makes up the tough, elastic fibers of scar tissue, and is the main structural material of our body‐bones, tendons, ligaments, and skin all contain collagen. This amino acid may significantly contribute to the development of embryo in mature period (dark color period).

Some marine invertebrates including mollusca, crustacean, and echinoderm have characteristic distribution patterns of free amino acids in which all the seafood commonly contain certain amounts of arginine, glycine, alanine, proline, and glutamic acid. Shrimps, lobsters, crabs, and shellfish contain larger amounts of glutamic acid, glycine, and alanine than fish does (Komata, 1990).

Ruiz‐Capillas and Moral (Ruiz‐Capillas & Moral, 2004) reported that FAAs are one of the most important fractions of non‐protein nitrogen in crustaceans. Many of these FAAs, such as alanine, glutamic acid, and glycine, are responsible for flavor and taste. Alanine and glycine have sweet tastes, and glutamic acid has the “umami” taste which is typical of crustaceans (Yamanaka & Shimada, 1996). FAAs have also been used as quality indices in various fish and crustacean species.

Dipeptides such as carnosine and anserine are the major dipeptides in vertebrates but not in invertebrates (Abe, 1995; Konosu, 1982; Van Waarde, 1988). As expected, these compounds were not detected in the muscle of freshwater prawn samples in this study.

### 3.3 Adenosine triphosphate (ATP) and its related compounds

Table 4 represents the ATP‐related compounds (μmole/g) of freshwater prawn in different gender and the egg. There were no significant differences in ATP‐related compounds between male meat (8.26 ± 1.57 μmole/g) and female meat (8.80 ± 2.44 μmole/g), majorly composed of ADP, ATP, AMP, and IMP. This value in egg sample (28.33 ± 2.27 μmole/g) was significantly higher than that of meat samples with the highest value belonging to IMP (18.48 ± 1.24 μmole/g). AMP took the second place in egg sample with value being 5.44 ± 0.53 μmole/g, while inosine and hypoxanthine ranged from 1.40 ± 0.46 to 1.61 ± 0.07 μmole/g. Previous studies showed that IMP is considered as desirable flavor enhancer of the umami taste (Fuke, 1994; Komata, 1990; Lindsay, 1994) and AMP is also known as flavor‐active compound (Konosu, 1982).

**Table 4:** ATP‐related compounds (μmole/g) of male meat, female meat, and egg of freshwater prawn

ATP‐related compounds (μmole/g)	Male meat	Female meat	Egg
ATP	1.77 ± 0.41^ab^	2.22 ± 0.89^a^	0.62 ± 0.22^b^
ADP	3.47 ± 0.47^a^	3.78 ± 1.22^a^	0.79 ± 0.20^b^
AMP	1.27 ± 0.26^b^	1.41 ± 0.14^b^	5.44 ± 0.53^a^
IMP	0.83 ± 0.44^b^	0.85 ± 0.32^b^	18.48 ± 1.24^a^
Hypoxanthine	0.33 ± 0.11^b^	0.51 ± 0.21^b^	1.40 ± 0.46^a^
Inosine	0.11 ± 0.13^b^	0.02 ± 0.01^b^	1.61 ± 0.07^a^
Total	8.26 ± 1.57^b^	8.80 ± 2.44^b^	28.33 ± 2.27^a^

Note

Tests were performed in triplicate; values are means ± standard deviation; mean values in the same column with different superscript letters are significantly different at *p* < 0.05.

Abbreviations: ATP, adenosine triphosphate; ADP, adenosine diphosphate; AMP, adenosine monophosphate; IMP, inosine 5’‐monophosphate.

It is well established that after death of fish and shellfish, ATP is enzymatically degraded by the following pathway: ATP→ADP→AMP→IMP→Inosine→Hypoxanthine (Botta, 1994; Dépêche, Gilles, Daufresne, & Chiapello, 1979; Konosu, 1982). ATP degradation is one of the most important biochemical changes in the postmortem muscle of fish and shellfish. This process has long been recognized as an accurate way to evaluate freshness of fish and shellfish products (Hong, Regenstein, & Luo, 2017).

Nucleotides have been considered to be taste enhancers and potentiators. In combination with other compounds such as glutamic acid, they produce a flavor synergistic effect. In particular, IMP, as one of ATP breakdown compounds, is desirable flavor enhancer associated with the umami taste of fish and shellfish, and it is formed as an intermediate in the degradation of nucleotide precursors. It reaches a peak concentration within one or two days of postmortem stage, and as it decreases in concentration, fish become less flavorful and less acceptable. Hypoxanthine represents the lowest compound measured in the adenine degradation scheme when nucleotides are measured relatively to fish quality. Hypoxanthine can contribute to fish bitterness. On the other hand, IMP is correlated with desirable sweet and salty tastes in fish (Botta, 1994).

### 3.4 The pH value, urea, and ammonia contents

The pH value, urea, and ammonia of freshwater prawn in different gender and egg prawn are shown in Table 5. There were no significant differences in pH value and ammonia between male and female samples, with the respective pH value being 7.04 ± 0.04 and 6.99 ± 0.04 and respective ammonia amount being 2.81 ± 0.44 and 2.60 ± 0.37 μmole/g. Urea was not detected in meat samples, while it was detected at high value in egg sample with 58.35 ± 7.77 μmole/g. Dépêche et al. (1979) revealed that the modifications in urea level can be related, at least partly, to the possibilities of urea formation through the urea cycle pathway. Relatively high amounts of amino acids and urea have been found in eggs and early embryos of the guppy *Poecilia reticulate* (Dépêche & Schoffeniels, 1975). In osmoregulation species, urea appears as an important osmotic effector already in the very early stages of the embryonic development (Price & Daiber, 1967; Read, 1971). In teleost fishes, urea is increasing in embryos upon acclimation of pregnant females to concentrated media. This ability to use urea as an osmotic effector is lost in adult fishes. The content of such free amino acids as arginine, ornithine, and aspartic acid plays an important role in the urea formation (Dépêche et al., 1979). Such a role has been demonstrated in the case of aspartate by Stubbs and Krebs (1975) and also by Briggs and Freedland (1976) in the mammalian hepatocytes.

**Table 5:** pH value, urea, and ammonia compounds (μmole/g) of male meat, female meat, and egg of freshwater prawn

	Male meat	Female meat	Egg
pH	7.04 ± 0.04^a^	6.99 ± 0.04^a^	6.50 ± 0.00^b^
Urea	–*	–	58.35 ± 7.77^a^
Ammonia	2.81 ± 0.44^b^	2.60 ± 0.37^b^	6.03 ± 2.21^a^

Note

Tests were performed in triplicate; values are means ± standard deviation; mean values in the same column with different superscript letters are significantly different at *p* < 0.05.

*Not detected.

## 4 CONCLUSIONS

The meat of freshwater prawn is a lean seafood (fat content of <5%) with high protein and free amino acid contents which are higher than those of prawn egg. There were no significant differences in biochemical characteristics between male and female prawns. This report provides useful and fundamental data to comprehend the biochemical characteristics of freshwater prawns.

## CONFLICT OF INTEREST

The authors declare that there is no conflict of interest.

## ETHICAL STATEMENT

This report does not conduct any human or animal tests.
